# Interface Shear Strength at Various Joint Types in High-Strength Precast Concrete Structures

**DOI:** 10.3390/ma13194364

**Published:** 2020-09-30

**Authors:** Young-Jin Kim, Won-Jong Chin, Se-Jin Jeon

**Affiliations:** 1 Department of Infrastructure Safety Research, Korea Institute of Civil Engineering and Building Technology, 283, Goyang-daero, Ilsanseo-gu, Goyang-si, Gyeonggi-do 10223, Korea; yjkim@kict.re.kr (Y.-J.K.); wjchin@kict.re.kr (W.-J.C.); 2 Department of Civil Systems Engineering, Ajou University, 206, Worldcup-ro, Yeongtong-gu, Suwon-si, Gyeonggi-do 16499, Korea

**Keywords:** interface shear strength, high-strength concrete, ultra-high-performance concrete, shear key, dry joint, wet joint, push-off test

## Abstract

More precast concrete structures have recently been constructed due to their many advantages when compared to conventional cast-in-place construction. Structural behavior at the joints between the precast segments can significantly affect the overall integrity, safety, and serviceability of the structure. In this study, therefore, the interface shear strength of high-strength precast members was investigated by performing push-off tests with the following variables: compressive strength of precast members, dry or wet joint, number and height of shear keys, joint width, filler type, curing temperature, and lateral compressive stress. The test results were analyzed to reveal the effect of each test variable on the joint shear strengths of the specimens. For instance, the failure loads were increased by 14–140%, depending on the lateral compressive stress, as the specified compressive strength of the precast members was increased from 80 to 150 MPa in the dry joints. The failure loads of the wet joints strongly depended on the strength of the filler rather than on that of the precast members and, as a result, the specimen with ultra-high-strength concrete filler was 46–48% stronger than those with high-strength mortar filler. The shear strengths of various joint types obtained from the test were further analyzed in comparison with the predictive equations of Japan Society of Civil Engineers (JSCE) and American Association of State Highway and Transportation Officials (AASHTO) with the aim of validating the appropriateness of these design provisions. In particular, an improved value of a coefficient in the JSCE equation is proposed to cover a range of compressive strengths in various precast members and filling materials.

## 1. Introduction

The strength of concrete has been greatly increased over the last few decades and high-strength concrete with a compressive strength over 55 MPa, according to ACI Committee 363, has become common [1]. In addition, the concept of high-performance or ultra-high-performance concrete, which combines various superior mechanical properties such as ductility, toughness, durability, and flowability, in addition to strength, has also been proposed and utilized in practice [2,3,4]. In addition, precast concrete construction has been increasingly preferred to conventional cast-in-place construction because of the higher plant-based quality control, safer and accelerated prefabricated on-site construction, etc. [5] Therefore, high-strength precast concrete structures have become a worldwide trend in the concrete industry [6,7,8]. In the precast-type construction, structural behavior at the joints between the precast segments or blocks can significantly affect the safety and serviceability of the entire structure. The joints can be divided into two types: wet, where the joint has a certain space filled by casting a selected material in-place, and dry, where the match-cast surfaces of the two segments are in direct contact with the optional application of epoxy between the surfaces. This epoxy is known to increase the joint strength and watertightness. In both types of joints, shear keys are basically used to increase the load-carrying capacity, especially for shear, and to accommodate the assembly of segments. The structural integrity at the joints has been investigated experimentally and analytically with a focus on the cracking pattern and failure mode of the shear keys [9,10,11,12,13,14,15,16,17].

**Figure 1:**
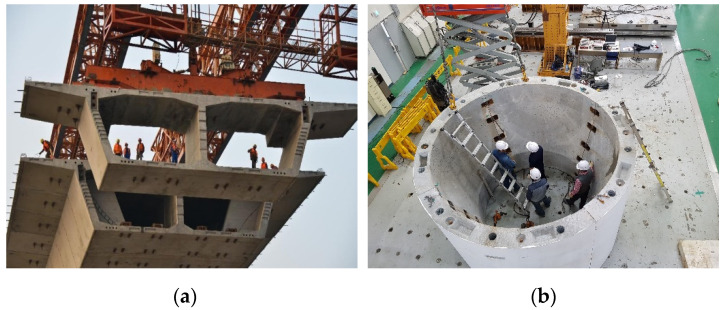
Examples of precast concrete construction: (**a**) prestressed concrete box-girder bridge; and (**b**) wind tower [18].

Figure 1 shows two representative examples of precast concrete construction where dry joints were used with a number of shear keys. Prestressing tendons that penetrate the segments are often used, not only for introducing compressive stresses to concrete according to the concept of prestressed concrete and for ensuring connection of the precast segments, but also for increasing frictional resistance at the joint, as investigated in this study.

In particular, special attention should be paid to the joints of the high-strength precast members because they may be weaker than the members themselves, thereby degrading the integrity and continuity of a structure, unless proper measures are taken at the joints. In this study, therefore, the structural behavior of the joints, focusing on the interface shear strength of the high-strength precast members, was investigated by performing push-off tests. The test variables were compressive strength of precast members, dry or wet joint, number and height of shear keys, joint width, filler type, curing temperature, and lateral compressive stress. The test results were analyzed to reveal the effect of each variable on the joint shear strengths of the specimens. Furthermore, the results were compared with two representative predictive equations for the interface shear strength to validate the appropriateness of these design provisions. Finally, an improved form of the predictive equation was proposed to cover a wide range of compressive strengths, including high strength and ultra-high strength, in precast members and fillers.

## 2. Test Specimens and Variables

### 2.1. Test Specimens

Two kinds of specified concrete compressive strength were employed, as shown in Table 1, to attain high strength and ultra-high strength precast members, 80 and 150 MPa in terms of a cylinder specimen with the dimensions of 100 mm × 200 mm (diameter × height), respectively. The mixture with 150 MPa strength was also intended for ultra-high-performance concrete. The steel fibers contained in the 150 MPa mixture had diameter of 0.2 mm and a length of 19.5 or 16.3 mm. The tensile strength of the fiber was more than 2000 MPa. Although the precast members with a compressive strength of as high as 180 MPa were tested via a similar procedure in a study [19], the strength was extended to a lower range in our study to cover various real circumstances and conditions that can be encountered in a construction project. The joint types of the specimens were divided into dry joint and cast-in-place wet joint, where the dry joint was either epoxy-glued or not and the wet joint had a few types of filler cast into it, as detailed in the test variables. The term “wet joint” describes a cast-in-place joint and “dry joint” a match-cast joint, regardless of the epoxy application, to avoid confusion herein. The epoxy-glued match-cast joint is also called the wet joint in some studies.

**Figure 2:**
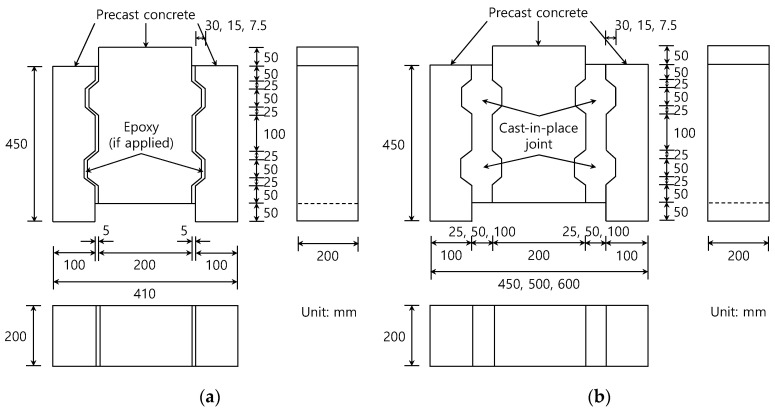
Dimensions of test specimens with two shear keys: (**a**) dry joint; and (**b**) wet joint.

Figure 2 representatively shows the details of the specimens for each joint type with two shear keys. In the case of one shear key, it was located at the middle height. The shear key shapes conformed to the recommendations in the AASHTO and JSCE specifications [20,21,22]. For example, the ratio of height to width in the shear key was less than 0.5 to enhance the strength of the shear key part with a desirable failure mode. Although the shear key height of 30 mm was included, heights shallower than 30 mm were also tested to investigate the improved performance of high-strength materials. The interface surfaces of the precast specimens remained smooth without any treatment, as typical in precast members. However, in the case of the interface between cast-in-place segments, the shear strength can be increased by proper surface treatment such as waterjet to roughen the surface. The strength can further be increased by the contribution of the fibers contained in fiber-reinforced concrete [23]. For the epoxy-coated dry joints, the thickness was limited to 5 mm and an average compressive stress of 0.28 MPa was applied using a temporary prestressing system until the epoxy was cured to realize the full bond and a consistent thickness [21]. The compressive and shear strengths of the epoxy were 77 and 24.5 MPa, respectively, at 7 days.

**Figure 3:**
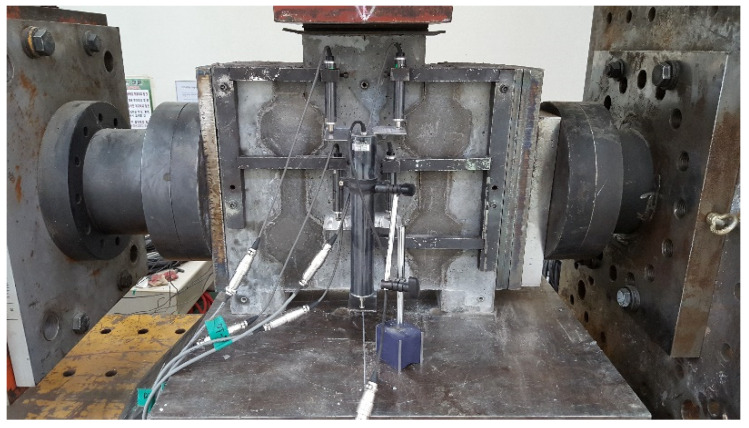
Set-up of push-off test specimens.

The set-up of the push-off test specimens is shown in Figure 3. Lateral loads were applied on the loading plates at both sides using a horizontal actuator to simulate the lateral compressive stress, which was one of the test variables, and maintained while the vertical load was applied for push-off. In actual construction processes, prestressing tendons usually penetrate a series of precast segments and interfaces, as shown in Figure 1. In the case of the wet joints, the lateral load was introduced after the filler had reached the specified compressive strength through a specified curing period. The vertical load was applied on a loading plate with dimensions of 200 mm × 200 mm × 30 mm, thus converting the concentrated load to a uniformly distributed load applied on the extruded upper face of the specimen, up to the ultimate stage at the loading rate of 0.4 mm/min. Then, half of the vertical load was applied at each joint. Although concrete strain gauges and displacement gauges were installed on the specimens, any analysis of the measured strains and displacements was beyond the scope of our study, in which the failure loads and shear strengths were the primary concern.

### 2.2. Test Variables

**Figure 4:**
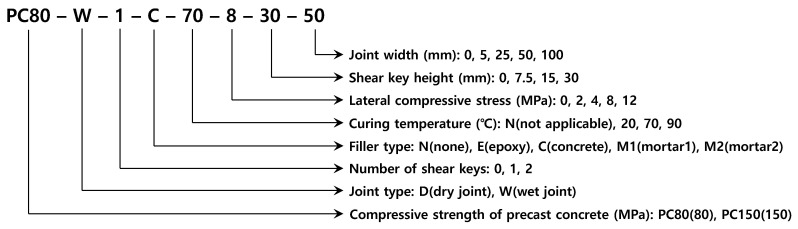
Specimen identification.

The test variables are shown in Table 2 and Table 3 for the precast members with compressive strengths of 80 and 150 MPa, respectively. They include such parameters as dry or wet joint, number and height of shear keys, joint width, filler type, curing temperature, and lateral compressive stress. The compressive strength of the precast members was also a test variable. Each specimen in Table 2 and Table 3 was identified by the abbreviations explained in Figure 4. In the dry joint, the effect of the epoxy on the strength was examined. The difference in strength between the precast member and the filler of the wet joint must be minimized to retain the overall structural efficiency. Therefore, the compressive strength of the filler ranged from 60 to 150 MPa, in line with the high or ultra-high strength of the precast members. The number and height of the shear keys were considered as variables to examine how they affect the failure mode and ultimate capacity in terms of interface shear. Zero in the number of shear keys indicates the joint with a flat interface. The available curing temperature depends on the condition of a site and a high steam curing temperature can lead to rapid strength development. Curing temperatures of 90 and 70 °C were used to simulate full steam curing and ordinary heat curing, respectively, and of 20 °C to correspond to room temperature, as applied for usual curing without any special measures. Various lateral compressive stresses were applied to investigate the various prestresses provided by the prestressing tendons. Although the width of the wet joint was set to 50 mm in the previous relevant study [19], we treated it as a variable in the range from 25 to 100 mm.

## 3. Analysis of Test Results

### 3.1. Failure Mode

**Figure 5:**
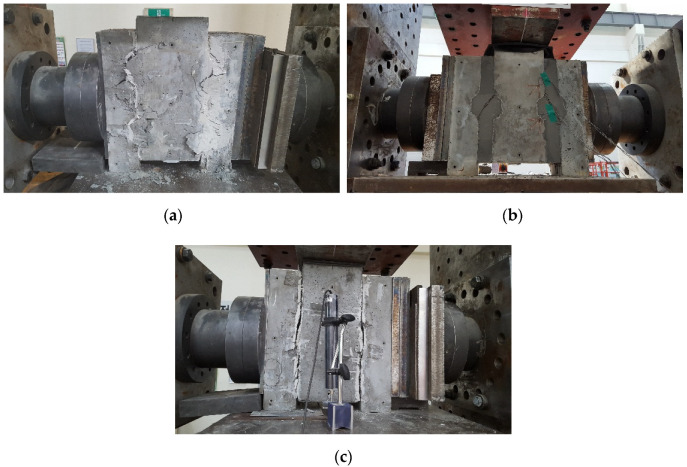
Failure shape of specimens: (**a**) multiple cracks (PC80-D-2-E-N-8-30-5); (**b**) diagonal cracks and shear-off (PC80-W-1-C-90-8-30-50); and (**c**) interface debonding (PC80-D-1-E-N-2-7.5-5).

The cracking patterns and failure modes of the specimens varied depending on the test variables, and they were closely related to each specimen’s ultimate strength. In particular, the joint type, shear key height, and lateral compressive stress strongly affected the cracking pattern. In the epoxy-glued dry joint with relatively tall shear keys and high lateral compressive stress, diagonal cracks were initiated at the lower face of the shear key. However, the cracks subsequently propagated in multiple ways, as shown in Figure 5a: some propagated in a diagonal direction in both the male and female shear key parts, while others developed in a vertical direction, resulting in shear-off failure of the shear key base. This type of multiple cracking mode is very desirable because the specimen behaved as a monolithic specimen without any joint, fully utilizing its capacity without any weaker part. On the other hand, in the wet joint with relatively tall shear keys, the diagonal cracks initiated at the two corners of a shear key were connected together, ending in the sloped shear-off failure of the wet joint that is shown in Figure 5b, even though the lateral compressive stress was not large. The abovementioned two corners were subjected to high stress concentration considering the configuration of the keyed wet joint. When the cracks occurred in an ultra-high-performance precast concrete member or filler, the bridging effect of the steel fibers contributed to resisting crack opening. In the case of no shear key or relatively shallow shear keys, the failure surface tended to form along the interface, as presented in Figure 5c, where some cases exhibited simultaneous crushing of the material at the lower sloped contact face. This type of interface debonding could also be observed in the tall shear keys if the frictional resistance provided by the lateral compressive stress was not sufficient.

### 3.2. Dry Joint

#### 3.2.1. Number of Shear Keys

**Figure 6:**
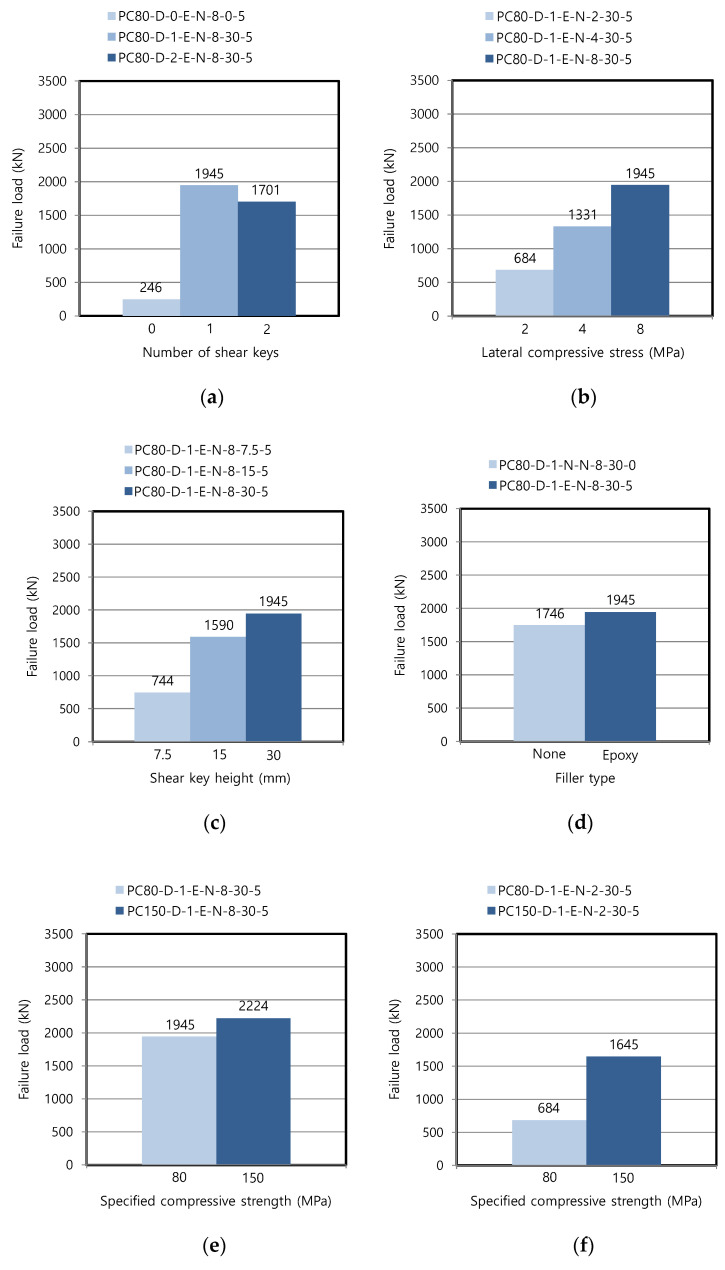

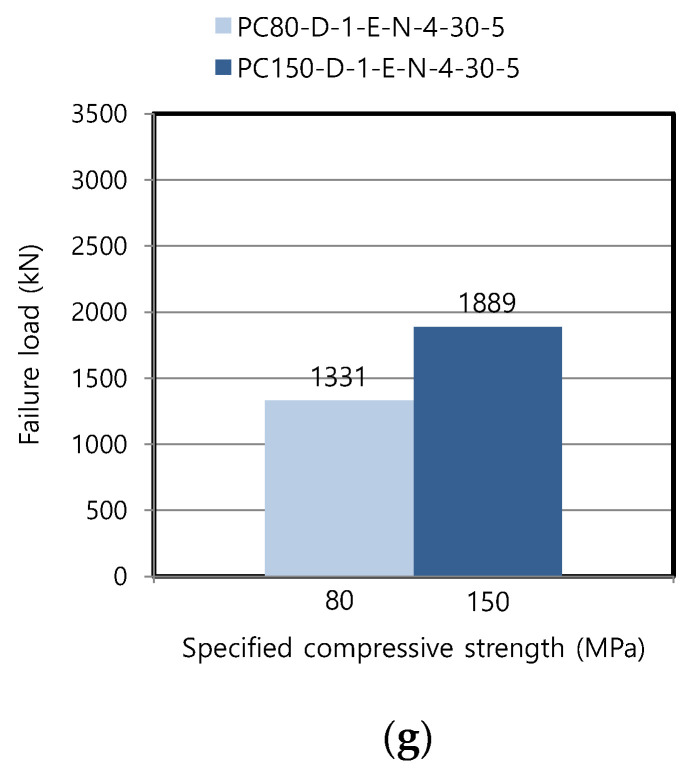
Failure loads in dry joint for each variable: (**a**) number of shear keys; (**b**) lateral compressive stress; (**c**) shear key height; (**d**) filler type; (**e**) compressive strength (Case 1); (**f**) compressive strength (Case 2); and (**g**) compressive strength (Case 3).

The failure load in the following graphs indicates the ultimate load, i.e., the maximum load measured in the vertical actuator. Although the presence of shear keys increased the failure load, the number of shear keys did not have a noticeable effect on the load in the epoxy-glued condition, as shown in Figure 6a. This can be attributed to the development of multi-directional cracks, as presented in Figure 5a, irrespective of the number of shear keys. In comparison, the failure load was proportional to the number of shear keys in the previous study [19], where the compressive strength of precast member was as high as 180 MPa. It seems that the weakened strength of the precast member with 80 MPa in the present study induced multiple cracks in the main body, which made the number of shear keys less relevant to the failure load despite the increased interface area in two shear keys. In general, a number of shear keys are provided at the interface of actual precast segments, as shown in Figure 1.

#### 3.2.2. Lateral Compressive Stress

The failure loads were increased as the lateral compressive stress applied perpendicular to the interface was increased because of the additional frictional resistance, as shown in Figure 6b. However, the increasing trend of the failure loads was not linear with respect to the magnitude of the lateral compressive stress. Therefore, a sufficient compressive stress needs to be provided along the interface in a real structure to resist the shear stress induced by design loads.

#### 3.2.3. Height of Shear Key

The failure loads were increased as the shear key height increased, as shown in Figure 6c. It was revealed that the specification of the minimum 30 mm height of a shear key [20,21,22] empirically derived from normal strength concrete was still effective even for the high-strength range.

#### 3.2.4. Presence of Epoxy

Figure 6d shows that epoxy coating increased the failure load by 11.4% for the specimen with one shear key, thus providing additional shear strength to the resistance of the shear key. The increase was 3.5% for the specimen with two shear keys (PC80-D-2-N-N-8-30-0: 1643 kN and PC80-D-2-E-N-8-30-5: 1701 kN). The AASHTO specifications [21] prevented the use of the dry joints without epoxy to establish the multiple protection system for prestressing tendons. This study revealed that the epoxy coating also contributed to increasing the load-carrying capacity.

#### 3.2.5. Compressive Strength of Precast Concrete

The failure loads were increased by 14.3% as the specified concrete compressive strength was increased from 80 to 150 MPa, with the lateral compressive stress maintained at 8 MPa, as presented in Figure 6e. Figure 6f,g shows that the increase was even as much as 140% and 41.9% for the lateral compressive stresses of 2 and 4 MPa, respectively. These results imply that the failure mechanism of the epoxy-glued dry joint was also related to the failure of the precast parts adjacent to the joint. Referring to Figure 5a, this type of failure did not occur along the interface, due to the high level of bonding provided by the epoxy along with lateral compressive stress. The interface shear strength at this type of joint was sufficiently high that the failure was strongly affected by the strength of the precast members, and this increase was higher for the lower lateral compressive stress. Therefore, the higher lateral compressive stress can compensate for the relatively low concrete compressive strength of precast members in the shear strength at the epoxied dry joint. The previous study [19] performed the same type of test using precast members with a specified compressive strength of 180 MPa. The failure load was further increased to 2387 kN for the specimen corresponding to PC180-D-1-E-N-8-30-5 and, therefore, followed a similar trend for the compressive strength.

### 3.3. Wet Joint

#### 3.3.1. Number of Shear Keys

**Figure 7:**
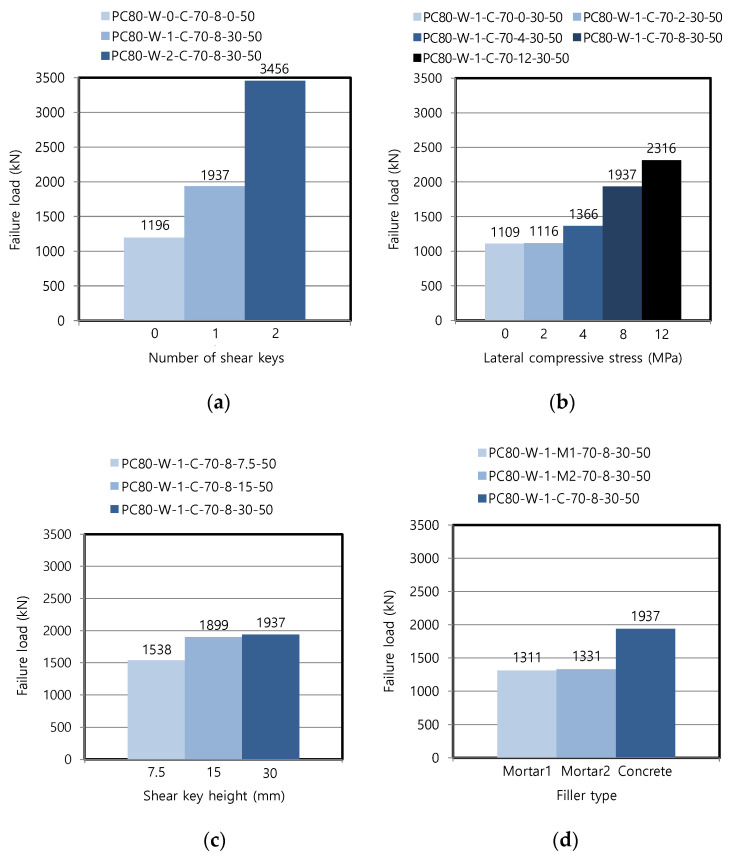

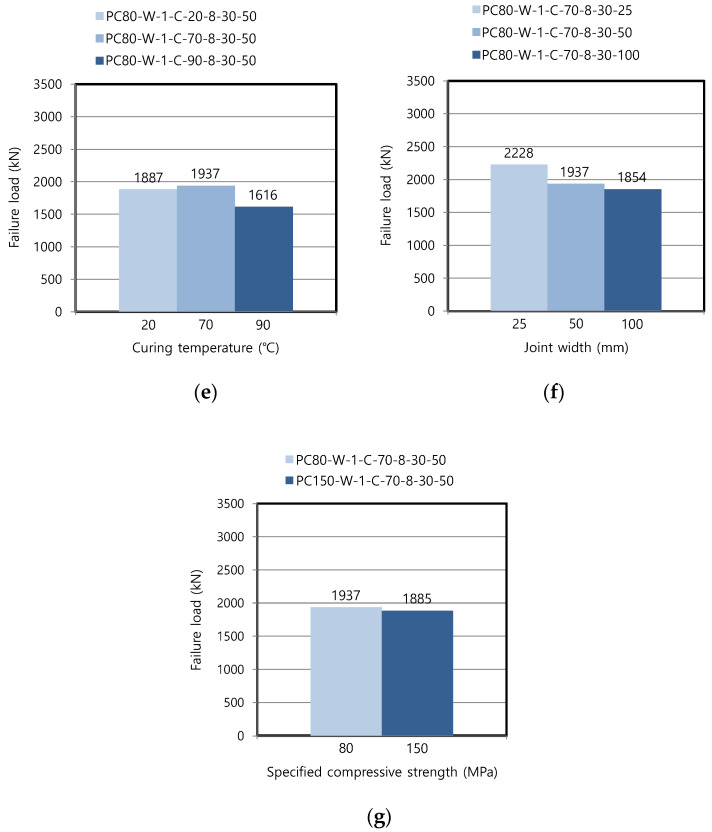
Failure loads in wet joint for each variable: (**a**) number of shear keys; (**b**) lateral compressive stress; (**c**) shear key height; (**d**) filler type; (**e**) curing temperature; (**f**) joint width; and (**g**) compressive strength.

Figure 7a shows that the failure loads were increased with increasing number of shear keys in the wet joint. This result was expected because the final failure mode was the diagonal cracks in each shear key part, as shown in Figure 5b for one shear key. Because the failure occurred only at the joint made with concrete of 150 MPa compressive strength, and not at the precast member of 80 MPa compressive strength, the overall failure loads were higher than those of the dry joints, as discussed in Section 3.2.1. Understandably, the flat joint without any shear key exhibited the lowest failure load because cohesion and friction were the only load-resisting mechanism in this case, without any geometrical contribution of the shear keys to the shear strength.

#### 3.3.2. Lateral Compressive Stress

The range of the lateral compressive stress was extended in the wet joint, compared to that of the dry joint, in order to investigate the effect of the lateral force on shear strength in more detail. The failure loads were increased as the lateral compressive stress was increased, as shown in Figure 7b, similar to the trend of the dry joint, although the trend was not exactly linear. As discussed below, the equation of JSCE [22,24] incorporated this nonlinear relationship between the failure loads and lateral compressive stress.

#### 3.3.3. Height of Shear Key

Although the failure loads were increasingly affected by the shear key height in Figure 7c, this increase was less than that of the dry joint shown in Figure 6c. The similar results for the shear key height of 15 and 30 mm were caused by the same failure mode of diagonal cracks connecting the two corners (see Figure 5b), in which the shear key height is less relevant to this failure mode.

#### 3.3.4. Filler Type

The failure loads depended on the strength of the filler, as shown in Figure 7d. The failure load of the specimen with the concrete filler having an ultra-high compressive strength of 150 MPa was 45.5–47.7% higher than that of the specimens with the mortar filler having a compressive strength of 60–70 MPa, which was attributed to the occurrence of the failure at the wet joint part with the cracks not propagating to the precast members with 80 MPa strength, as representatively shown in Figure 5b, in all the cases of Figure 7d.

It was reported that the ultra-high-performance concrete filler with 180 MPa strength was very effective in increasing the shear strength of the wet joint between the precast members with the same compressive strength [19]. The failure took place at the joint itself, as in the present study. In another study [25], a cast-in-place ultra-high-performance concrete filler with 158 MPa strength applied to the shear-keyed joint of precast concrete members with 61 MPa strength showed superior performance to any other fillers. The precast concrete part cracked with an intact wet joint, which demonstrated the excess strength of the filler. The results of these two previous studies and of the present study indicate that the filler strength needs to be equal to or higher than that of the precast member in order to prevent the wet joint from being the weak point. In this respect, an ultra-high-strength concrete filler can generally be a better choice than other materials for enhancing the structural efficiency.

#### 3.3.5. Curing Temperature

High-temperature steam curing is often employed for rapid strength development of precast concrete, considering an efficient manufacturing process at a plant. However, the available curing temperature can often be limited after casting the filling material into the wet joints of a structure due to inferior on-site conditions. According to a previous study [26], the specified compressive strength of ultra-high-performance concrete can eventually be attained, even at a curing temperature lower than 90 °C, i.e., that of steam curing, if the moisture is continuously supplied for a sufficient curing duration. This condition was realized for the specimens with the wet joint by placing them in a chamber controlled with 100% relative humidity. As a result, the failure loads did not vary significantly for the different curing temperatures in Figure 7e. The slightly lower failure load at 90 °C than that at other temperatures was possibly caused by side effects arising from less than optimal steam curing process [26].

#### 3.3.6. Joint Width

Figure 7f shows that the failure loads were decreased as the joint widths were increased in this series. Although the decrease was not significant, the joint width may have affected the stress distribution related to the typical diagonal cracks of the wet joint, as shown in Figure 5b. However, observing the test results of other series, the decreasing trend of the failure loads could not be generalized because some series showed a similar tendency, while some other series exhibited an ambiguous tendency: for example, the failure loads of PC80-W-2-M1-70-8-30-25, PC80-W-2-M1-70-8-30-50, and PC80-W-2-M1-70-8-30-100 were 2005, 1793, and 1889 kN, respectively. Overall, the width of the wet joint did not seem to have a significant effect on the failure load if the relative strength of the joint filler was sufficient compared to that of the precast part.

#### 3.3.7. Compressive Strength of Precast Concrete

The failure of the specimens with the wet joint largely depended on the strength of the joint filler, as mentioned in Section 3.3.4. Therefore, the compressive strength of the precast members did not significantly affect the interface shear strength, which only varied by 2.7%, as shown in Figure 7g. The specimens with another filler also revealed similar failure loads: 1311 and 1272 kN (3.0% difference) for PC80-W-1-M1-70-8-30-50 and PC150-W-1-M1-70-8-30-50, respectively. These results contrast with the trend of the dry joint shown in Figure 6e–g because of the different failure modes.

### 3.4. Comparison between Dry Joint and Wet Joint

Comparison of Figure 6b and Figure 7b shows that, given the same compressive strength of precast members, the shear strength of the wet joint can exceed that of the epoxied dry joint if the filler strength is higher than that of the precast members. This phenomenon was dominant under the relatively low lateral compressive stress of 2 or 4 MPa, whereas the shear strengths of the dry and wet joints became similar at the high lateral compressive stress of 8 MPa.

However, given the identical strengths of the precast part and filler, the epoxied dry joint showed a higher shear strength than that of the wet joint: the failure load of the dry joint PC150-D-1-E-N-8-30-5 (2224 kN) was 18.0% higher than that of PC150-W-1-C-70-8-30-50 (1885 kN). This trend matched that in the previous study [19], where the strengths of the precast part and filler were 180 MPa. In summary, an epoxy-coated dry joint is preferred to a wet joint because its interface shear strength is generally higher than that of the wet joint cast by the filler having a strength equivalent to or weaker than that of the precast part. The wet joint, using a filler stronger than the precast part, can be structurally more efficient than the epoxied dry joint, which introduces some limitation in choosing the filler. Another potential issue is the low constructability of the wet joint which involves careful mixing and curing of the filler at the site.

## 4. Applicability of Design Equations

### 4.1. General Remarks

Many predictive equations for the interface shear strength at the joints of concrete members have been proposed, some of which have been reflected in the design provisions. However, the validity of these equations needs to be examined for concrete of high to ultra-high strength because they were mostly derived from the tests of normal strength concrete. The shear transfer mechanism incorporated in these equations basically consists of friction and cohesion. The effect of the shear keys on the interface shear strength can be included in both the friction and cohesion terms of the equations. The contribution of the shear keys can be accounted for in two different approaches in terms of the geometry: the shear key geometry is directly considered in some design equations [20,22,24,27] and is indirectly reflected as a very rough surface or an indented surface in some other equations [21,28,29]. To investigate the contribution of the shear keys in more detail, the former types of equation were analyzed for comparison with the experimental data, the representative of which are presented in AASHTO [20] and JSCE [22,24].

Equation (1) shows the design equation intended for the dry joints without epoxy, which was specified in the AASHTO guide specifications for segmental concrete bridges [20]. The first term accounts for the contribution of shear keys and the second incorporates the frictional resistance with the friction coefficient of 0.6. However, because this type of joint is no longer allowed according to the AASHTO specifications [21], Equation (1) has become less useful. Nevertheless, Equation (1) was compared with the shear strengths obtained from the specimens with the dry joint without epoxy.

(1) $$V_{nj}=A_{k}\sqrt{0.006792f_{c}^{\prime }}\left(12+2.466f_{pc}\right)+0.6A_{sm}f_{pc},$$

where $V_{nj}$ is the nominal joint shear capacity (*N*), $A_{k}$ is the area of the base of all keys in the failure plane (mm^2^), $f_{c}^{\prime }$ is the compressive cylinder strength of concrete (MPa), $f_{pc}$ is the compressive stress in concrete (MPa), and $A_{sm}$ is the area of contact between smooth surfaces on the failure plane (mm^2^).

On the other hand, Equation (2) has been employed mainly in Japan for both general concrete [22] and ultra-high-strength concrete [24]. However, because the JSCE specifications [22] state that the provisions presented are effective only when the characteristic (specified) compressive strength does not exceed 80 MPa, it is questionable whether Equation (2) can also be utilized to specify higher-strength concrete. This issue is revisited in Section 4.3.

(2) $$V_{cw}=\mu f_{cd}^{\prime }{}^{b}\sigma_{nd}{}^{1-b}A_{cc}+0.1A_{k}f_{cd}^{\prime },$$

where $V_{cw}$ is the shear strength (*N*), μ is the average friction coefficient of solid contact (0.45), $f_{cd}^{\prime }$ is the design compressive cylinder strength (MPa), $\sigma_{nd}$ is the average compressive stress which acts perpendicular to shear plane (MPa), $A_{cc}$ is the area of shear plane in compression zone (mm^2^), b is the coefficient indicating plane configuration (0–1 and 0.5 in the case of the joint of precast members with adhesive agent), and $A_{k}$ is the area of compressive side of shear key (mm^2^). Equation (2) features the coefficient b that can be adjusted depending on the joint type. Although 0.5 was only suggested for a joint with epoxy in the specifications, other researchers proposed coefficients other than 0.5 for different types of joint: 0.4 [30] and 0 [31] for the wet joint and the dry joint without epoxy, respectively. Furthermore, a recent study [19] proposed a value of b more optimized for the epoxied dry joint of ultra-high-performance concrete. Equation (2) therefore has the versatility to accommodate various types of joint.

Note that the shear capacity or shear strength in Equations (1) and (2) is half the failure load measured in the push-off test specimen of the present study.

### 4.2. Comparison with the AASHTO Equation

**Figure 8:**
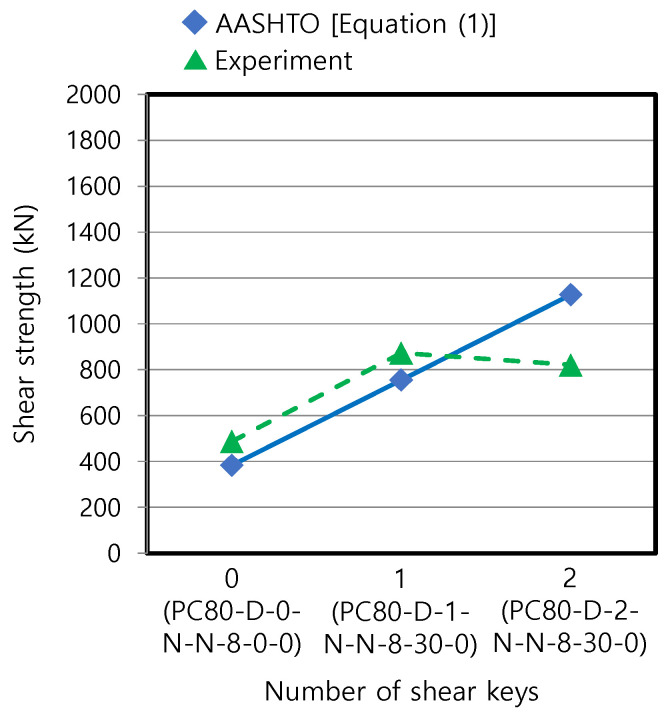
Comparison of test data with predictive equation of AASHTO [20] (dry joint without epoxy).

Equation (1) originally proposed for a dry joint without epoxy was compared with the test results of the corresponding three specimens. They satisfy the minimum shear key height of 30 mm in AASHTO [20,21]. $A_{k}$ in Equation (1) is 0, 20,000, and 40,000 mm^2^ for zero, one, and two shear keys, respectively, and $A_{sm}$ is 80,000, 60,000, and 40,000 mm^2^ for zero, one, and two shear keys, respectively, referring to the dimensions of the specimens shown in Figure 2. The AASHTO equation underestimated the shear strengths for zero and one shear key by 21.4% and 13.4%, respectively, while it overestimated the strength for two shear keys, as shown in Figure 8. Although Equation (1) applied a linear relation between the shear strength and the number of shear keys, the measurement for the two shear keys was not linear, possibly due to some errors introduced during the test. The underestimation was also observed in a previous study [19] for the specimen with higher strength (180 MPa), equivalent to PC180-D-1-N-N-8-30-0 in our study.

### 4.3. Comparison with the JSCE Equation

The specimens that met the 30 mm requirement of the minimum shear key height in JSCE [22] were mainly investigated for consistency. When applying Equation (2), $\sigma_{nd}$ was considered without 50% reduction for additional safety as recommended in the JSCE specifications [22]. In addition, the specified (characteristic) compressive strength was used for $f_{cd}^{\prime }$ for a reasonable comparison with the test data, although $f_{cd}^{\prime }$ originally refers to the design compressive strength obtained according to the concept of limit state design [22]. From now, various joint types were analyzed by changing the coefficient b.

**Figure 9:**
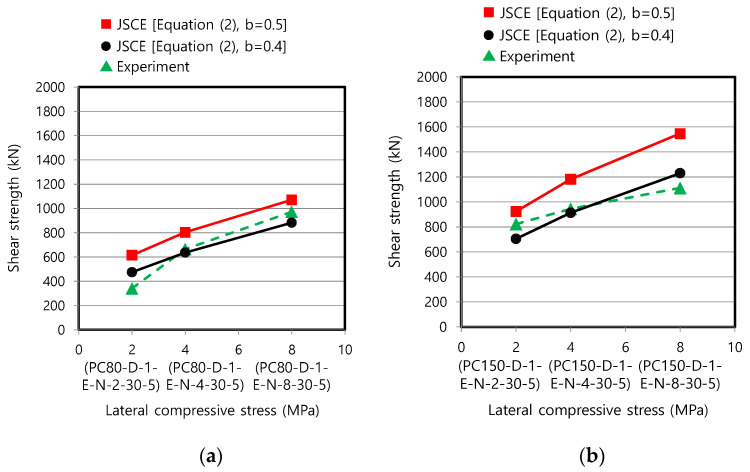
Comparison of test data with predictive equation of JSCE [22,24] (dry joint with epoxy): (**a**) specimens with $f_{c}^{\prime }$ = 80 MPa; and (**b**) specimens with $f_{c}^{\prime }$ = 150 MPa.

First, the dry joint with epoxy was analyzed (Figure 9) by applying both b = 0.5 in the original specifications [22] and an improved value of b = 0.4 that was proposed as particularly suitable for ultra-high-performance concrete with a strength of 180 MPa [19]. The form of the JSCE equation implies that the shear strength is not in linear relation with respect to the concrete strength and lateral compressive stress. Figure 9 shows that the JSCE equation significantly overestimates the test data when using b = 0.5, as already reported in the previous study [19]: overestimation of the shear strengths by 10.1–79.9% and 12.3–39.1% for the specimens with $f_{c}^{\prime }$ = 80 and 150 MPa, respectively. However, by lowering the coefficient to 0.4, the differences were greatly reduced to −9.2% to 38.9% and −14.3% to 10.6%, where the minus sign indicates the underestimation, for the specimens with $f_{c}^{\prime }$ = 80 and 150 MPa, respectively (Figure 9). An appropriate degree of structural strength underestimation is desirable in design to ensure conservative and safer structures. The test results reconfirm that the revised value of b = 0.4 is acceptable for evaluating the interface shear strength when the concrete strength of precast members falls within the high to ultra-high strength range, as previously reported in the relevant study [19]. That is, this study revealed that Equation (2) can also be applied to concrete structures with a compressive strength of more than 80 MPa, which was the original limitation [22], on the condition that the amended value of b is used. To completely remove the unsafe cases presented in Figure 9, the coefficient needs to be further reduced, but this may induce excessive conservatism.

However, the actual design of the joint when using Equation (2) incorporates a few additional safety factors, including the material factor for concrete, member factor, and 50% reduction of $\sigma_{nd}$. Therefore, it is highly probable that Equation (2) also underestimates the shear strengths if these measures for safety margins are additionally employed, even when using the original version of b = 0.5. The aforementioned discussion is also effective in the subsequent analysis of the wet joint.

**Figure 10:**
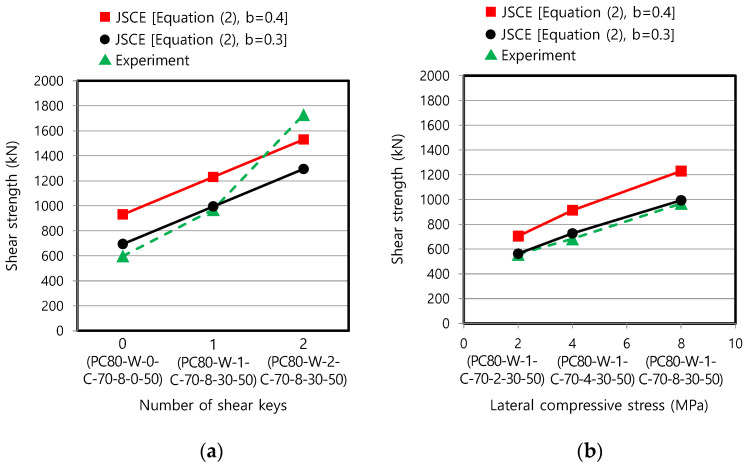
Comparison of test data with predictive equation of JSCE [22,24] for each variable (wet joint with ultra-high-strength concrete filler): (**a**) number of shear keys; and (**b**) lateral compressive stress.

Secondly, the applicability of Equation (2) was also examined for the wet joint in Figure 10. No consensus has been reached on which strength should be used in Equation (2) between the precast member strength and the filling material strength when they differ. However, we consider it more reasonable to use the strength of the joint filler because the failure occurred at the wet joint, as demonstrated in Figure 5b. The specimens with the ultra-high-strength concrete filler were representatively analyzed, as shown in Figure 10. The coefficient b = 0.4 proposed for the wet joint in an earlier study [30] did not show good agreement with the test data as considerable overestimation was evident. Therefore, a reduced coefficient b = 0.3 was applied for the wet joint in this study and gave much better agreement than that for b = 0.4. The theoretical and test values only differed by 0.9–6.5%, as shown in Figure 10b.

In both the dry joint with epoxy and the wet joint, we conclude that the first term of Equation (2) that includes $f_{cd}^{\prime }$ is overestimated, resulting in inaccurate estimation of the shear strength, in the range of both high and ultra-high strength, if the conventional values of b are adopted. However, the equation with the reduced values of b better approximated the test data in both cases.

Finally, the dry joint without epoxy was analyzed with b = 0 proposed earlier [31] in Equation (2), which means that the frictional resistance is completely affected solely by the lateral compressive stress, irrespective of the concrete strength. The JSCE equation underestimated the shear strengths of the specimens already analyzed in Figure 8 by as much as 26.0–48.7%, which was far less accurate than the AASHTO equation. Therefore, the JSCE equation does not seem appropriate for evaluating a dry joint without adhesive agent, especially with high to ultra-high-strength concrete, although this approach was used in a previous study [31].

## 5. Conclusions

Precast concrete construction using high and even ultra-high-strength concrete has become increasingly popular, replacing conventional cast-in-place concrete construction. The interface shear strength at the joints of these precast members is of primary importance to ensure the overall structural integrity, safety, and serviceability. Therefore, the shear strength and failure mechanism of various joint types typically used in high to ultra-high strength precast concrete members were investigated by push-off tests in this study. Based on the experimental results, the following conclusions can be drawn:

1. In dry joints, the test variables were compressive strength of precast members, number and height of shear keys, presence of epoxy, and lateral compressive stress. The cracks propagated in multiple directions through the male and female shear key parts in the epoxy-glued dry joint with relatively tall shear keys and high lateral compressive stress. Similar to the behavior of normal strength concrete, the failure loads were increased as the height of shear key and lateral compressive stress were increased, due to the geometrical contribution of the shear keys and frictional resistance, respectively. The minimum shear key height of 30 mm specified in some provisions remained effective in high-strength concrete. The epoxy coating also contributed to the increase in the failure load. The compressive strength of the epoxied precast members significantly affected the failure load because the cracks occurred not along the interface but in the members themselves, and this trend was intensified at relatively low lateral compressive stress.
2. The wet joints were tested with the following variables: compressive strength of precast members, number and height of shear keys, joint width, filler type, curing temperature, and lateral compressive stress. The failure loads were increased as the number and height of shear keys, and lateral compressive stress were increased. The curing temperature of the ultra-high-strength concrete filler was not a crucial factor provided that the moisture was continuously supplied for a sufficient curing duration. The width of the wet joint did not show an explicit trend in regard to the failure load. The wet joint with relatively tall shear keys tended to crack in a diagonal direction at the joint itself, leading to sloped shear-off failure. Therefore, the failure loads strongly depended on the strength of the filler rather than on that of the precast members and, as a result, the specimen with ultra-high-strength concrete filler was 46–48% stronger than those with high-strength mortar filler. In general, the strength of the filler needs to be equivalent to or higher than that of the precast member in order to prevent the wet joint from being the weak point.
3. Two representative predictive equations for the interface shear strength at the joints specified in the AASHTO and JSCE specifications were compared with the test data. In particular, the validity of these equations, which were originally derived from the tests of normal strength concrete, was examined to extend their applicability to the range of high and ultra-high-strength concrete. In applying the JSCE equation for the epoxied dry joint and the wet joint, the term that includes the exponential function of the concrete strength greatly overestimated the shear strength in the range of high to ultra-high strength, resulting in an overall overestimation of the shear capacity, when using the conventional values of the exponent. Therefore, we have proposed revising the JSCE equation by reducing the exponent to some extent when using this equation to evaluate the epoxied dry joint or the wet joint in high to ultra-high strength precast members or filling materials. The improved JSCE equation reasonably predicted the trend of the shear strengths obtained from the test. However, the shear strength of the dry joint without epoxy could not be properly estimated by using either the AASHTO or JSCE equation in high-strength precast members.

## Figures and Tables

**Table 1:** Mix proportion of precast concrete. Unit: kg/m^3^

$f_{c}^{\prime }\;{}^{1}$ (MPa)	W ^2^	C ^3^	BS ^4^	FA ^5^	SF ^6^	FP ^7^	FA ^8^	CA ^9^	SHA ^10^	EA ^11^	AD ^12^	SF ^13^
80	165	420	189	56	35	-	674.3	827.9	4.2	-	9.1	-
150	176.4	793	99.1	-	99.1	237.9	872.3	-	7.9	39.7	19.8	117

^1^ Specified concrete compressive strength; ^2^ water; ^3^ cement; ^4^ blast furnace slag; ^5^ fly ash; ^6^ silica fume; ^7^ filling powder; ^8^ fine aggregate; ^9^ coarse aggregate; ^10^ shrinkage reducing agent; ^11^ expansive agent; ^12^ admixture (superplasticizer); ^13^ steel fiber.

**Table 2:** Variables of push-off test ($f_{c}^{\prime }$ = 80 MPa).

Joint Type	Number of Shear Keys	Filler Type	Curing Temperature (°C)	Lateral Compressive Stress (MPa)	Height of Shear Key (mm)	Width of Joint (mm)	Specimen ID
Dry Joint	1	Epoxy	-	2	30	5	PC80-D-1-E-N-2-30-5
					15	5	PC80-D-1-E-N-2-15-5
					7.5	5	PC80-D-1-E-N-2-7.5-5
				4	30	5	PC80-D-1-E-N-4-30-5
					15	5	PC80-D-1-E-N-4-15-5
					7.5	5	PC80-D-1-E-N-4-7.5-5
				8	30	5	PC80-D-1-E-N-8-30-5
					15	5	PC80-D-1-E-N-8-15-5
					7.5	5	PC80-D-1-E-N-8-7.5-5
		None		8	30	0	PC80-D-1-N-N-8-30-0
	2	Epoxy		8	30	5	PC80-D-2-E-N-8-30-5
					15	5	PC80-D-2-E-N-8-15-5
					7.5	5	PC80-D-2-E-N-8-7.5-5
		None		8	30	0	PC80-D-2-N-N-8-30-0
	0	Epoxy		8	0	5	PC80-D-0-E-N-8-0-5
		None		8	0	0	PC80-D-0-N-N-8-0-0
Wet Joint (cast-in-place)	1	Concrete ^1^	90	8	30	25, 50, 100	PC80-W-1-C-90-8-30-25(50, 100)
			70	0	30	50	PC80-W-1-C-70-0-30-50
				2	30	50	PC80-W-1-C-70-2-30-50
				4	30	50	PC80-W-1-C-70-4-30-50
				8	30	25, 50, 100	PC80-W-1-C-70-8-30-25(50, 100)
					15	25, 50, 100	PC80-W-1-C-70-8-15-25(50, 100)
					7.5	50	PC80-W-1-C-70-8-7.5-50
				12	30	25, 50, 100	PC80-W-1-C-70-12-30-25(50, 100)
			20	8	30	25, 50, 100	PC80-W-1-C-20-8-30-25(50, 100)
					15	25, 50, 100	PC80-W-1-C-20-8-15-25(50, 100)
					7.5	50	PC80-W-1-C-20-8-7.5-50
		Mortar 1 ^2^	70	8	30	25, 50, 100	PC80-W-1-M1-70-8-30-25(50, 100)
					15	25, 50, 100	PC80-W-1-M1-70-8-15-25(50, 100)
					7.5	50	PC80-W-1-M1-70-8-7.5-50
		Mortar 2 ^3^			30	25, 50, 100	PC80-W-1-M2-70-8-30-25(50, 100)
					15	25, 50, 100	PC80-W-1-M2-70-8-15-25(50, 100)
					7.5	50	PC80-W-1-M2-70-8-7.5-50
	2	Concrete	70	8	30	25, 50, 100	PC80-W-2-C-70-8-30-25(50, 100)
					15	50	PC80-W-2-C-70-8-15-50
					7.5	50	PC80-W-2-C-70-8-7.5-50
		Mortar 1			30	25, 50, 100	PC80-W-2-M1-70-8-30-25(50, 100)
					15	50	PC80-W-2-M1-70-8-15-50
					7.5	50	PC80-W-2-M1-70-8-7.5-50
		Mortar 2			30	50	PC80-W-2-M2-70-8-30-50
					15	50	PC80-W-2-M2-70-8-15-50
					7.5	25, 50, 100	PC80-W-2-M2-70-8-7.5-25(50, 100)
	0	Concrete		2	0	50	PC80-W-0-C-70-2-0-50
				4		50	PC80-W-0-C-70-4-0-50
				8		25, 50, 100	PC80-W-0-C-70-8-0-25(50, 100)
		Mortar 1		2	0	50	PC80-W-0-M1-70-2-0-50
				4		50	PC80-W-0-M1-70-4-0-50
				8		25, 50, 100	PC80-W-0-M1-70-8-0-25(50, 100)
		Mortar 2		2	0	50	PC80-W-0-M2-70-2-0-50
				4		50	PC80-W-0-M2-70-4-0-50
				8		25, 50, 100	PC80-W-0-M2-70-8-0-25(50, 100)

^1^ Specified compressive strength = 150 MPa (ultra-high-performance concrete; refer to Table 1 for mix proportion); ^2^ specified compressive strength = 60 MPa (high-strength mortar); ^3^ specified compressive strength = 70 MPa (high-strength mortar).

**Table 3:** Variables of push-off test ($f_{c}^{\prime }$ = 150 MPa).

Joint Type	Number of Shear Keys	Filler Type	Curing Temperature (°C)	Lateral Compressive Stress (MPa)	Height of Shear Key (mm)	Width of Joint (mm)	Specimen ID
Dry joint	1	Epoxy	-	2	30	5	PC150-D-1-E-N-2-30-5
				4	30	5	PC150-D-1-E-N-4-30-5
				8	30	5	PC150-D-1-E-N-8-30-5
Wet joint (cast-in-place)	1	Concrete ^1^	70	8	30	25, 50, 100	PC150-W-1-C-70-8-30-25(50, 100)
		Mortar 1 ^2^			30	25, 50, 100	PC150-W-1-M1-70-8-30-25(50, 100)
		Mortar 2 ^3^			30	25, 50, 100	PC150-W-1-M2-70-8-30-25(50, 100)

^1^ Specified compressive strength = 150 MPa (ultra-high-performance concrete; refer to Table 1 for mix proportion); ^2^ specified compressive strength = 60 MPa (high-strength mortar); ^3^ specified compressive strength = 70 MPa (high-strength mortar).
